# Aberrant accumulation of phosphorylated BRCA1 in brainstem-type and cortical-type Lewy bodies in Lewy body disease

**DOI:** 10.1093/jnen/nlaf004

**Published:** 2025-02-05

**Authors:** Masataka Nakamura, Aya Murakami, Dennis W Dickson, Yusuke Yakushiji

**Affiliations:** Department of Neurology, Kansai Medical University, Hirakata, Osaka, Japan; Department of Neurology, Kansai Medical University, Hirakata, Osaka, Japan; Department of Neuroscience, Mayo Clinic, Jacksonville, FL, United States; Department of Neurology, Kansai Medical University, Hirakata, Osaka, Japan

**Keywords:** α-synuclein, BARD1, BRCA1, DNA damage response, Lewy body disease

## Abstract

BRCA1 plays important roles in several biological events during the DNA damage response (DDR). We aimed to determine whether cytoplasmic accumulation of BRCA1 or its phosphorylated form, pBRCA1, is specific to cytoplasmic inclusions in tauopathies, or if it also occurs in α-synuclein-positive inclusions in Lewy body disease (LBD). Using brain tissue from pure LBD, LBD with Alzheimer disease (AD) co-pathology (LBD-AD), and control cases, the immunohistochemical distributions of BRCA1, pBRCA1, its binding partner BARD1, and 53BP1 were examined. The results showed that pBRCA1 (Ser1423) and BARD1 accumulated in brainstem-type Lewy bodies (LBs), whereas only pBRCA1 (Ser1423) was present in cortical-type LBs. There was no significant difference in the frequency of pBRCA1 (Ser1423)-positive LBs between the pure LBD and LBD-AD cases. pBRCA1 (Ser1423) was minimally detected in neuronal nuclei in controls and was absent in neuronal nuclei in LBD cases. In control and LBD cases, 53BP1-immunoreactive deposits were present in the neuronal nuclei. Thus, DDR dysfunction due to cytoplasmic sequestration of pBRCA1 (Ser1423) may play a role in LBD pathogenesis. Additionally, the selective accumulation of BARD1 in brainstem-type LBs, but not cortical-type LBs, points to distinct mechanisms in the formation of these inclusion types, offering further insights into LBD pathology.

## INTRODUCTION

Parkinson disease (PD) is a neurodegenerative disorder characterized by the presence of Lewy bodies (LBs) and Lewy neurites (LNs), both composed of α-synuclein (α-syn).^1^^,^^2^ LBs are also found in PD with dementia (PDD) and dementia with LBs (DLB), collectively termed Lewy body disease (LBD).^3^ LBD can be divided into brainstem (BLBD), transitional (TLBD), and diffuse type (DLBD) types, based on the distribution of LBs.^4^ There are 2 types of LBs, ie, brainstem-type LBs, which are eosinophilic masses possessing a dense core and a peripheral halo on hematoxylin and eosin (H&E) staining, and cortical-type LBs, which are poorly defined eosinophilic structures without a conspicuous halo or core.

Terminally differentiated, post-mitotic neurons are highly active cells with very restricted regeneration potential.^5^ Their high metabolic activity produces large amounts of reactive oxygen species (ROS), which can lead to double-strand breaks (DSBs) in neuronal DNA. DSBs are mainly repaired by the homologous recombination (HR) and non-homologous end-joining (NHEJ) pathways.^6^ The choice between these 2 pathways depends on DNA-end resection,^7^ regulated by the opposing actions of breast cancer type 1 susceptibility protein (BRCA1) and p53-binding protein 1 (53BP1). BRCA1, along with its partner protein BRCA1-associated ring domain 1 (BARD1), promotes DNA-end resection and HR whereas 53BP1 restricts the resection and promotes NHEJ.^8^ Although HR is typically absent in neurons, recent evidence suggests that it may be active in neuronal DNA repair.^9^

In PD, significant upregulation of the histone H2AX (γ-H2AX), a marker of DSB damage, has been reported.^10^^,^^11^ In addition, proteins encoded by PD-related genes have been shown to regulate DNA repair. For instance, nuclear α-syn promotes DSB repair via NHEJ.^12^ Endogenous leucine-rich repeat kinase 2 (LRRK2) promotes HR repair,^13^ and DJ-1 interacts with a key protein involved in NHEJ repair.^14^ These 3 proteins are components of LBs,^15^ suggesting that mutation-induced loss of function or sequestration within LBs may lead to the accumulation of DNA damage and defective DNA repair in LBD.

We and other groups have reported that BRCA1 and phosphorylated BRCA1 (pBRCA1) accumulate in the phosphorylated tau inclusions in Alzheimer disease (AD).^16^^,^^17^ In AD, extracellular amyloid β (Aβ) induces DNA damage and upregulates BRCA1 expression in neurons.^18^ However, BRCA1 is mislocalized to the cytoplasm, where it colocalizes and co-aggregates with phosphorylated tau, rendering it dysfunctional. To date, there has been limited investigation of BRCA1-immunoreactivity in LBD.^17^ Given that LB pathology is commonly observed in AD and LBD often presents with some degree of concomitant Alzheimer-type pathology, we speculate that BRCA1 dysfunction may also play an important role in the pathogenesis of LBD. The extent of this dysfunction may vary depending on the presence or absence of Aβ.

In the present study, we aimed to clarify the differences in BRCA1 dysfunction between AD and LBD and to investigate whether the presence of Aβ alters the underlying mechanisms of BRCA1 dysfunction in LBD. BRCA1 functions in DNA repair by forming a complex with BARD1. Therefore, we examined the distribution of immunoreactivities for BARD1 as well as for BRCA1. Additionally, in response to DNA damage, BRCA1 undergoes phosphorylation at specific sites. We used antibodies specific to pBRCA1 (Ser1423) and pBRCA1 (Ser1524) to investigate its phosphorylation status. Furthermore, to determine whether HR or NHEJ is preferentially activated, we also analyzed the immunoreactivity of 53BP1. We found that pBRCA1 (Ser1423) and BARD1 were accumulated within brainstem-type LBs, whereas only pBRCA1 (Ser1423) accumulated within cortical-type LBs. There was no difference in the subcellular localization of 53BP1 between control and LBD cases. These findings imply that pBRCA1 (Ser1423) dysfunction due to cytosolic mislocalization could play a significant role in the pathogenesis of LBD. These results highlight differences in LBs formation and DNA repair mechanisms, offering new insights into LBD pathophysiology.

## METHODS

### Human materials

Brain tissue was obtained from pure LBD (*n* = 8), LBD with AD co-pathology (LBD-AD) (*n* = 9), and control (*n* = 5) cases. Further information is provided in Table 1. These materials were obtained from the brain collection of the Department of Neurology at Kansai Medical University (*n* = 4) and the Mayo Clinic Jacksonville brain bank (*n* = 18). Neuropathological diagnosis of LBD was made according to current criteria.^4^ All cases underwent a standardized neuropathological assessment for Alzheimer-type pathology, as previously described.^19^ Braak neurofibrillary tangle (NFT) stage and Thal amyloid phase were assigned based on lesion counts in cortical and subcortical areas obtained using thioflavin S fluorescence microscopy. The neuropathological diagnosis of AD was based on consensus criteria.^20^ However, in this study, cases with low-, intermediate-, or high-likelihood were classified as LBD-AD. Consent for brain donation was obtained from next-of-kin in all cases, and all experimental procedures were conducted in accordance with the ethical guidelines set by Kansai Medical University.

**Table 1.** Characteristics of cases analyzed.

No	Pathological diagnosis	Clinical diagnosis	Age at death (y)	Sex	Braak stage	Thal phase	Race	Brain bank
1	TLBD	PDD	57	M	0	0	C	Mayo
2	TLBD	DLB	58	M	0	0	C	Mayo
3	DLBD	DLB	60	M	0	0	C	Mayo
4	TLBD	DLB	60	F	0	0	C	Mayo
5	DLBD	PDD	68	F	0	0	C	Mayo
6	TLBD	PDD	72	M	0	0	C	Mayo
7	TLBD	PDD	74	M	0	0	C	Mayo
8	TLBD	PDD	75	M	0	0	C	Mayo
9	DLBD	PDD	67	M	3	5	C	Mayo
10	DLBD	DLB	66	M	3	2	C	Mayo
11	DLBD	DLB	69	F	3	3	C	Mayo
12	DLBD	PD	70	F	3	4	C	Mayo
13	DLBD	PDD	72	M	2	2	J	Kansai
14	DLBD	PDD	81	F	3	1	C	Mayo
15	DLBD	PDD	85	M	3	3	C	Mayo
16	DLBD	DLB	86	M	3	3	J	Kansai
17	DLBD	PD	94	M	3	2	C	Mayo
18	Normal	Normal	59	F	0	0	C	Mayo
19	Normal	Normal	63	M	0	0	C	Mayo
20	Normal	Normal	65	F	0	0	J	Kansai
21	Normal	Normal	68	M	0	0	C	Mayo
22	Normal	Normal	69	M	0	0	J	Kansai

Abbreviations: C, Caucasian; DLB, Dementia with Lewy bodies; DLBD, Lewy body disease, diffuse type; F, female; J, Japanese; M, male; PD, Parkinson disease; PDD, PD with dementia; TLBD, Lewy body disease, transitional type.

### Immunohistochemistry

Formalin-fixed, paraffin-embedded, 5-μm-thick sections from the midbrain, amygdala, and temporal cortex were processed for immunohistochemistry. The sections were deparaffinized by immersion in xylene and hydrated by passage through graded ethanol solutions. The sections were steamed in distilled water or 10 mM citrate buffer (pH 6.0) for 30 minutes for antigen retrieval and then immunostained with mouse monoclonal antibody against BRCA1 (1:250, ab16780; Abcam, Cambridge, MA, USA), rabbit polyclonal antibody against pBRCA1 (Ser1423, 1:200, ab47325; Abcam), rabbit polyclonal antibody against pBRCA1 (Ser1524, 1:100, ab47276; Abcam), rabbit polyclonal antibody against BARD1 (1:100, ab115477; Abcam), rabbit polyclonal antibody against 53BP1 (1:1000, Abcam), or mouse monoclonal antibody against phosphorylated α-syn (p-α-syn; 1:1000, clone #64, No. 015-25191, Wako, Osaka, Japan), diluted with Tris-buffered saline-Tween 20 (TBS-T, 0.05%) containing 5% normal goat serum (NGS). Incubation was carried out overnight at 4° C. Consecutive serial sections were stained as follows: The first of the serial sections was stained with H. & E. or immunolabeled with anti-p-α-syn antibody. The second section was then stained with anti-pBRCA1 (Ser1423), anti-pBRCA1 (Ser1524), anti-BARD1, or anti-53BP1 antibodies to assess whether these proteins were present within the aggregates composed of α-syn. Primary antibodies were visualized with the appropriate Vectastain Elite ABC kits (Vector Laboratories, Burlingame, CA, USA), and 3,3’-diaminobenzine tetrahydrochloride was used as the chromogen. The severity of the p-α-syn- and BRCA1-, pBRCA1 (Ser1423)-, or BARD1-positive LBs were counted per power field and scored by using a semiquantitative grading system: absent = 0, scant = 1, sparse = 2, moderate = 3, or frequent = 4. The results are summarized in Table 2.

**Table 2.** Summary of semiquantitative scoring of neuropathological features in Lewy body disease.

No	Study groups	Brainstem-type LBs				Cortical-type LBs			
		p-α-syn	pBRCA1 (Ser1423)	BARD1	pBRCA1 (Ser1423)/p-α-syn (%)	p-α-syn	pBRCA1 (Ser1423)	BARD1	pBRCA1 (Ser1423)/p-α-syn (%)
1	Pure LBD	4	3	2	62.5	4	4	0	56.7
2	Pure LBD	4	3	2	58.8	4	4	0	72.5
3	Pure LBD	4	2	2	56.3	3	2	0	46.2
4	Pure LBD	4	4	3	100	4	4	0	79.5
5	Pure LBD	2	2	2	75.0	4	3	0	51.7
6	Pure LBD	2	1	1	57.1	1	1	0	60.0
7	Pure LBD	2	2	1	77.8	4	4	0	56.4
8	Pure LBD	2	2	2	100	4	4	0	59.6
9	LBD-AD	3	3	2	100	4	3	0	57.1
10	LBD-AD	4	4	2	83.3	4	4	0	82.1
11	LBD-AD	4	3	2	55.6	4	4	0	97.2
12	LBD-AD	2	2	2	75.0	4	4	0	58.3
13	LBD-AD	2	2	2	85.7	4	3	0	59.1
14	LBD-AD	2	2	2	87.5	4	4	0	67.6
15	LBD-AD	4	4	3	88.2	4	4	0	52.6
16	LBD-AD	4	4	2	76.0	4	4	0	70.6
17	LBD-AD	4	3	2	69.2	4	4	0	51.4

LBs,  Lewy bodies; AD, Alzheimer disease; LBD, Lewy body disease.

Double immunofluorescence staining of selected sections was performed. The following primary antibodies were used: mouse monoclonal antibody against p-α-syn (1:500, clone #64: Wako Chemicals) and rabbit polyclonal antibody against pBRCA1 (Ser1423, 1:100, ab47325; Abcam) or BARD1 (1:50, ab47276; Abcam). These primary antibodies were detected with Alexa Fluor 488-labeled goat anti-mouse IgG (1:200, Molecular Probes, Eugene, OR, USA) and Alexa Fluor 546-labeled goat anti-rabbit IgG (1:200, Molecular Probes), respectively. The slides were mounted with VECTASHIELD mounting medium (Vector Laboratories) and observed with an LSM 710 META confocal laser scanning microscope (Carl Zeiss AG, Oberkochen, Germany).

We assessed the staining specificity by replacing the primary antibodies with an appropriate amount of nonimmune rabbit serum or TBS-T solution containing 5% NGS, or by preincubating the primary antibodies with an excess of peptide immunogen. No reaction product deposits were observed in the sections thus treated.

### Semiquantitative analysis

To evaluate the proportion of p-α-syn-positive brainstem-type and cortical-type LBs that are immunoreactive for pBRCA1 (Ser1423) in cases of pure LBD and LBD-AD, midbrain and amygdala sections from all 17 LBD cases were analyzed. As mentioned above, the first serial section was immunolabeled with anti-p-α-syn antibody, and 5 distinct areas were selected from each section. For the substantia nigra, 5 distinct regions, including the ventral tegmental area, dorsolateral, dorsomedial, ventrolateral, and ventromedial, were selected. For the amygdala, 5 distinct regions were randomly selected from both medial and lateral areas. After counting immunopositive brainstem-type LBs in the substantia nigra and cortical-type LBs in the amygdala, the second sections were stained with anti-pBRCA1 (Ser1423) antibody. Immunopositive brainstem-type LBs in the substantia nigra and cortical-type LBs in the amygdala from the same neurons of the same 5 distinct areas for each section were then counted. The percentages of LBs immunoreactive for pBRCA1 (Ser1423) were calculated for each section and average percentages for both brainstem-type and cortical-type LBs were determined. All LB counts were performed at ×20 magnification by a single observer (A.M.). Statistical analysis was performed using the Mann-Whitney U test.

## RESULTS

### Brainstem-type LBs

**Figure 1.**
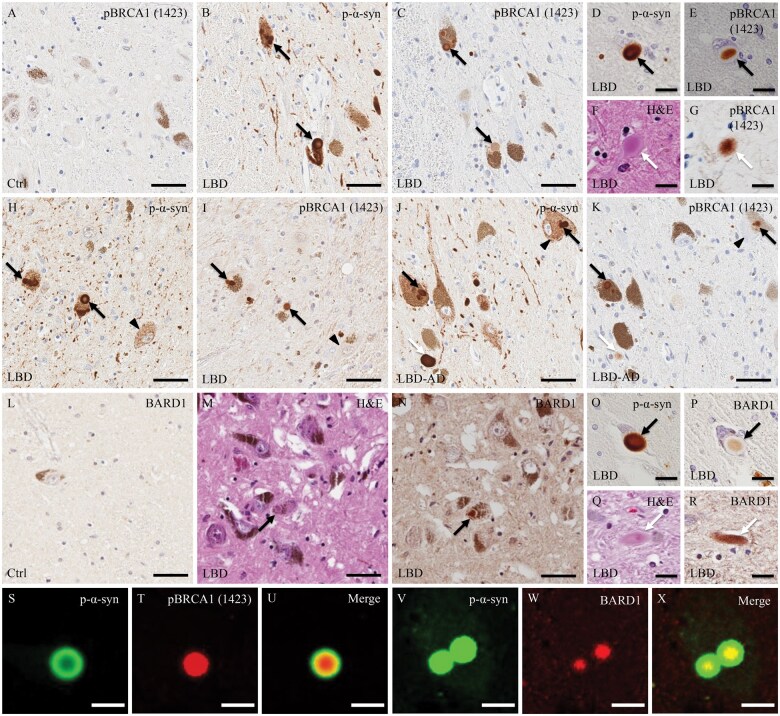
Immunohistochemical and double immunofluorescence analysis of pBRCA1 (Ser1423) and BARD1 in the substantia nigra of control and Lewy body disease (LBD) cases. (A) In the control case, slight deposits of pBRCA1 (Ser1423) immunoreaction product are seen in the nuclei of dopaminergic neurons in the substantia nigra, whereas the cytoplasm of these cells is completely devoid of immunoreactivity. (B-E) Serial sections immunostained for phosphorylated α-synuclein (p-α-syn) and pBRCA1 (Ser1423) show that p-α-syn-positive brainstem-type Lewy bodies (LBs; black arrows) are also reactive with anti-pBRCA1 (Ser1423) antibody. (F, G) Lewy neurites (LNs) (white arrows) identified by hematoxylin and eosin (H&E) staining are immunoreactive for pBRCA1 (Ser1423). (H-K) Serial sections show that both brainstem-type LBs (black arrows) and LNs (white arrows) are immunopositive for pBRCA1 (Ser1423) in LBD and LBD with Alzheimer disease co-pathology (LBD-AD) cases. In contrast, p-α-syn-positive diffuse cytoplasmic deposits (arrowheads) are immunonegative for pBRCA1 (Ser1423). LNs (white arrows) show less pronounced labeling of pBRCA1 (Ser1423) than p-α-syn. (L) BARD1-immunoreactivity is absent in the control case. (M-P) Brainstem-type LBs (black arrows) identified by H&E staining or p-α-syn-immunoreactivity are positive for BARD1 in LBD specimens. (Q, R) LNs (white arrows) identified by H&E are also immunopositive for BARD1. (S-X) Double immunofluorescence labeling for p-α-syn (S; green), pBRCA1 (1423) (T; red), and merged images (U), and that for p-α-syn (V; green), BARD1 (W; red), and merged images (X) in midbrain sections from LBD patients. pBRCA1(1423) is co-localized with p-α-syn in a brainstem-type LB. BARD1 is also co-localized with p-α-syn-immunopositive brainstem-type LBs. Scale bars: A-C, H-N = 50 μm; D-G, O-X = 10 μm.

In brain samples from controls, slight deposits of pBRCA1 (Ser1423) immunoreactive product were observed in the neuronal nuclei of the substantia nigra, whereas the cytoplasm of these cells was completely devoid of immunoreactivity (Figure 1A). In all LBD cases, pBRCA1 (Ser1423)-immunoreactivity was observed in brainstem-type LBs of the substantia nigra but not in neuronal nuclei (Figure 1B-E and H-K). The staining patterns varied, being uniform throughout the LBs, ring-like at the periphery, or concentrated in the central core of the LBs. Conversely, p-α-syn-immunoreactive granular inclusions were negative for pBRCA1 (Ser1423). As for LNs, the extent of pBRCA1 (Ser1423) labeling was less pronounced than that of p-α-syn (Figure 1F, G, J, and K). BARD1-immunoreactive deposits were not seen in the neurons or glial cells of the substantia nigra of controls (Figure 1L). BARD1-immunoreactivity was also observed in brainstem-type LBs and LNs (Figure 1M, N), but the proportions were low. BARD1-immunoreactivity was mainly localized to the central regions of brainstem-type LBs (Figure 1O-R). Double immunofluorescence labeling for p-α-syn and for either pBRCA1 (Ser1423) or BARD1 showed co-localization of p-α-syn-immunoreactivity with that of pBRCA1 or BARD1 within brainstem-type LBs (Figure 1S-X).

**Figure 2.**
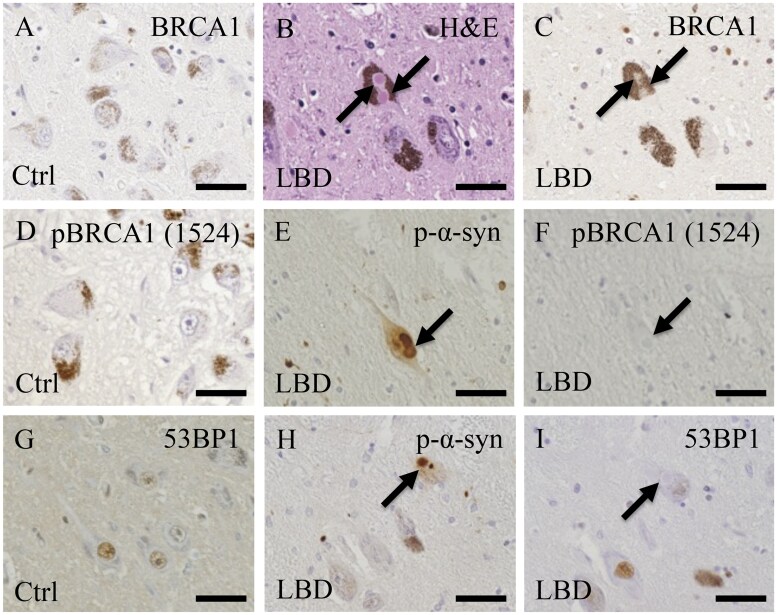
Immunohistochemical analysis of BRCA1, pBRCA (Ser1524), and BARD1 in the substantia nigra of control and Lewy body disease (LBD) cases. (A) In the control case, BRCA1-immunoreactive deposits are not observed within the nuclei or cytoplasm of neurons and glial cells. (B, C) Brainstem-type Lewy bodies (LBs; black arrows) identified by hematoxylin and eosin (H&E) staining are not immunoreactive for BRCA1 in the LBD specimen. (D) pBRCA1 (Ser1524)-immunoreactive deposits are found in neurons or glial cells in the control case. (E, F) Serial sections immunostained for phosphorylated α-synuclein (p-α-syn) and pBRCA1 (Ser1524) show that p-α-syn-positive brainstem-type LBs (black arrows) are not immunoreactive with anti-pBRCA1 (Ser1524) antibody. (G-I) 53BP1-immunoreactive deposits are found in some neuronal and astroglial nuclei in the control and LBD cases but brainstem-type LBs (black arrows) are not labeled. Scale bars: A-I = 50 μm.

In both control and LBD cases, BRCA1-immunoreactive deposits were observed in less than 1% of neurons or glial cells in the substantia nigra (Figure 2A and C). pBRCA1 (Ser1524)-immunoreactive deposits were not detected in the nucleus or cytoplasm of the neurons and glial cells in the substantia nigra (Figure 2D and F). 53BP1-immunoreactive deposits were found in 5% to 40% of the neuronal or astroglial nuclei in the substantia nigra (Figure 2G and I). Serial sections showed that these antibodies did not label LBs or LNs (Figure 2B, C, E, F, H, and I).

### Cortical-type LBs

**Figure 3.**
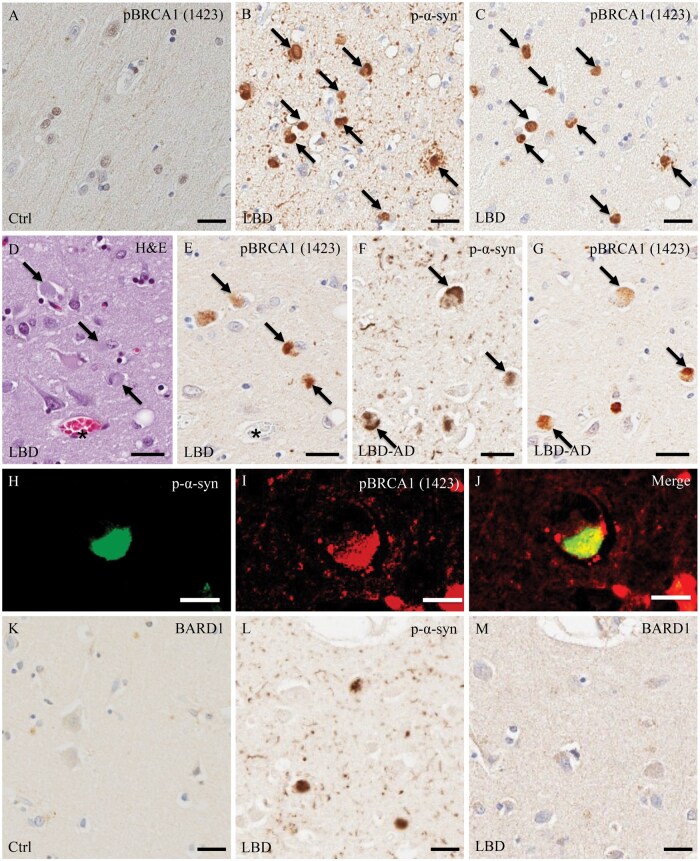
Immunohistochemical and double immunofluorescence analysis of pBRCA (Ser1423) and BARD1 in the amygdala and temporal cortex of control and Lewy body disease (LBD) cases. (A) In the control case, there is minimal pBRCA1 (Ser1423)-immunoreactivity in neuronal nuclei of the amygdala with no immunoreactivity in the cytoplasm. (B, C) Cortical-type Lewy bodies (LBs; black arrows) in the amygdala are immunoreactive for pBRCA1 (Ser1423) in LBD specimens. (D-G) Cortical-type LBs (black arrows) in the temporal cortex identified by H&E staining or p-α-syn immunohistochemistry are immunoreactive for pBRCA1 (Ser1423) in LBD and LBD with Alzheimer disease (LBD-AD) co-pathology cases. (H-J) Double immunofluorescence labeling for p-α-syn (H; green), pBRCA1 (1423) (I; red), and merged images (J) in amygdala sections from a LBD patient. pBRCA1(1423) is co-localized with p-α-syn in a cortical type LB. (K) BARD1-immunoreactivity is absent in the control case. (L, M) p-α-syn-immunopositive cortical LBs are negative for BARD1. A-C, amygdala; D-M, temporal cortex. Scale bars: A-G, K-M = 50 μm; H-J = 10 μm.

In control cases, pBRCA1 (Ser1423)-immunoreactivity was observed in the neuronal and glial nuclei of the amygdala and temporal cortex (Figure 3A). In all LBD cases, pBRCA1 (Ser1423)-immunoreactive deposits were observed in the cortical-type LBs of the amygdala and temporal cortex, but not in the neuronal nuclei (Figure 3B-G). Double immunofluorescence labeling for p-α-syn and pBRCA1 (Ser1423) showed co-localization of p-α-syn with pBRCA1-immunoreactivity within cortical-type LBs (Figure 3H-J). BARD1-immunoreactivity was not observed in neuronal nuclei or cytoplasm of the amygdala and temporal cortex in either control or LBD cases (Figure 3K-M).

**Figure 4.**
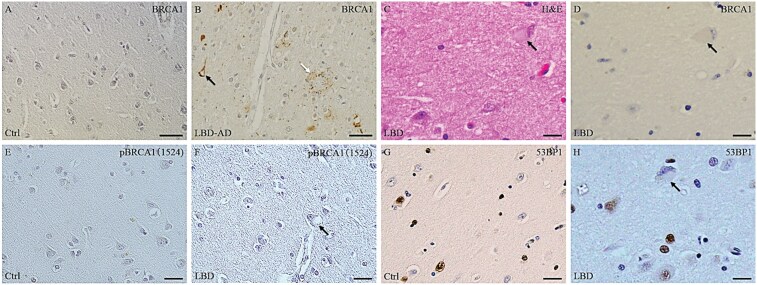
Immunohistochemical analysis of BRCA1, pBRCA (Ser1524), and BARD1 in the temporal cortex and amygdala of control and Lewy body disease (LBD) cases. (A) In the control case, BRCA1-immunoreactive deposits are not observed. (B) In the LBD with Alzheimer disease co-pathology (LBD-AD) case, BRCA1-immunoreactive deposits are observed in neurofibrillary tangles (NFTs, black arrows) and in dystrophic neurites (white arrows) surrounding neuritic plaques in the temporal cortex. (C, D) A Lewy body (LB) identified by H&E staining (black arrow) is immunonegative for BRCA1. (E, F) pBRCA1 (Ser1524)-immunoreactive deposits are not found in the control or LBD cases, including within LBs (black arrow). (G-I) 53BP1-immunoreactive deposits are found in some neuronal and astroglial nuclei in control and LBD cases, although 53BP-immunoreactivity is absent within the LB (black arrow). A, B, E, F temporal cortex; C, D, G, H, amygdala. Scale bars: A, B = 50 μm; C-F = 20 μm.

In control and pure LBD cases, BRCA1-immunoreactive deposits were not observed in neurons or glial cells (Figure 4A); however, as previously reported, strong BRCA1-immunoreactivity was detected in NFTs and dystrophic neurites in the amygdala and temporal cortex of LBD-AD cases (Figure 4B). However, serial sections showed that cortical-type LBs identified by H&E staining were immunonegative for BRCA1 (Figure 4C and D). In both control and LBD cases, pBRCA1 (Ser1524)-immunoreactivity was absent (Figure 4E and F), whereas 53BP1-immunoreactive deposits were present in 5% to 33% of the neuronal or astroglial nuclei (Figure 4G and H), with a higher percentage observed in LBD cases.

### Semiquantitative analyses

On semiquantitative analysis of the substantia nigra and amygdala from pure LBD and LBD-AD cases, the average percentages of pBRCA1 (Ser1423)-positive LBs among p-α-syn-immunoreactive brainstem-type and cortical-type LBs were 73.4 ± 17.1% and 60.3 ± 10.1%, and 80.1 ± 12.1% and 66.2 ± 14.2%, respectively. There were no significant differences between the pure LBD group and the LBD-AD group for pBRCA1 (Ser1423)-positive brainstem-type and cortical-type LBs, with U-statistics of 27.5 and 29.0, and p values of 0.44 and 0.54, respectively.

## DISCUSSION

To the best of our knowledge, this is the first report demonstrating the co-localization of pBRCA1 (Ser1423) and p-α-syn in both brainstem-type and cortical-type LBs. In addition, BARD1 accumulation was observed specifically within brainstem-type LBs. The distinct differences between these LBs suggest significant regional differences in their formation mechanisms. Importantly, there were no significant differences in the positivity rates of pBRCA1 (Ser1423) within LBs between pure LBD and LBD-AD cases. Although we have previously shown that pBRCA1 (Ser1423) is involved in the pathogenesis of AD,^16^ these findings suggest that the involvement of pBRCA1 (Ser1423) in the formation of LBs in LBD may be independent of the pathological processes associated with AD.

The process of LB formation consists of several stages.^21^^,^^22^ Diffuse cytoplasmic staining, the earliest observable abnormality related to α-syn accumulation, did not show immunoreactivity with the anti-pBRCA1 (Ser1423) antibody, suggesting that the incorporation of pBRCA1 (Ser1423) may occur at a later stage of LB formation, rather than during the early stages. However, the possibility of non-specific adherence of pBRCA1 (Ser1423) to aggregated α-syn deposits, as previously suggested, cannot be ruled out.^23^

BRCA1 is a nuclear-cytoplasmic protein implicated in several cellular pathways.^24^ In the nucleus, it plays roles in cell-cycle checkpoints, DNA repair, and transcriptional regulation, whereas in the cytoplasm, BRCA1 triggers apoptosis. Typically, BRCA1 levels are low or undetectable in resting (G0) cells but they increase during the late G1 and early S phases in proliferating cells.^25^ Following DNA damage, BRCA1 is rapidly hyperphosphorylated by ataxia-telangiectasia-mutated kinase (ATM), and its phosphorylation at Ser1423 is required for G2/M checkpoint activation.^26^ The present findings indicate that post-mitotic neurons in LBD aberrantly re-enter the cell cycle following DNA damage, consistent with previous studies.^27^ The G2/M transition is regulated by the Cdk1/Cyclin B complex.^28^ To induce cell-cycle arrest at G2/M, BRCA1 transcriptionally regulates the expression of both Wee1 kinase, an inhibitor of the Cdk1/Cyclin B complex, and the 14-3-3 family of proteins, which sequester this complex in the cytoplasm.^29^ Intense Cyclin B has been documented in brainstem LBs in LBD cases,^30^ and 14-3-3 proteins have also been detected in brainstem-type and cortical-type LBs of LBD patients,^31^^,^^32^ suggesting a block in the G2/M transition in LBD neurons. However, the present studies showed that pBRCA1 (Ser1423) was highly expressed but mislocalized to the cytoplasm. Given the apoptotic role of BRCA1 in the cytoplasm, this aberrant cytoplasmic accumulation of pBRCA1 (Ser1423) may contribute to neuronal cell loss resulting from disrupted cell cycling at the G2/M phase.

The BRCA1-BARD1 complex enhances its E3 ubiquitin ligase activity, which is crucial for Cyclin B degradation and G2/M checkpoint regulation.^33^ In the present study, pBRCA1 (Ser1423) and BARD1 were elevated and detectable in brainstem-type LBs in LBD, potentially impairing their enzymatic function and disrupting Cyclin B degradation, leading to its cytoplasmic accumulation. In contrast, in cortical-type LBs in LBD, only pBRCA1 (Ser1423) was elevated, with BARD1 absent. pBRCA1 (Ser1423) may fail to form a heterodimer with BARD1 due to sequestration by p-α-syn or due to unbalanced overexpression of pBRCA1 (Ser1423). Consequently, the accumulation of pBRCA1 (Ser1423) within inclusions could lead to a deficiency in its E3 ubiquitin ligase activity, disrupting the regulation of its other substrates.

DSBs are among the most lethal form of DNA damage. BRCA1 promotes HR during S/G2 phases of the cell cycle, whereas 53BP1 facilitates error-prone NHEJ, the predominant repair pathway in non-proliferating neurons.^14^ In AD, reduced neuronal BRCA1 impairs the repair of ROS-induced DSBs,^34^ and recent evidence suggests that neurons can use mRNA as a template for HR repair.^9^ In the current study, 53BP1 was present in the neuronal nuclei of both control and LBD cases. Whereas pBRCA1 (Ser1423) was present in the neuronal nuclei of control cases, it was absent and mislocalized to the cytoplasm in LBD cases. This mislocalization of nuclear pBRCA1 (Ser1423) may promote NHEJ over HR, potentially contributing to neuronal degeneration in LBD.

Recent studies have shown that BRCA1, including its phosphorylated form and BARD1, can translocate to mitochondria following mitochondrial damage and therefore may play an important role in regulating mitophagy.^35^ BRCA1 deficiency blocks mitophagy, leading to the accumulation of damaged mitochondria. Mitochondrial dysfunction is widely recognized as a key factor in the pathogenesis of LBD. The observed cytoplasmic sequestration of pBRCA1 (Ser1423) may indicate a disruption of BRCA1’s mitochondrial functions, particularly in relation to mitophagy.

There are several limitations to this study. First, since the sample size was relatively small and nearly all patients were Caucasian, further research is needed to determine whether our findings are generalizable to other LBD patients. Second, the anatomical subdivisions examined may differed between cases, which could influence the observed findings and their interpretation.

In conclusion, the present study provides the first evidence of abnormal accumulation of pBRCA1 (Ser1423) in LBs in LBD, suggesting that its cytosolic sequestration can cause dysfunction of DNA damage response (DDR) mechanisms, ultimately leading to neurodegeneration. Our findings would be reinforced for example by evidence obtained from methods such as Western blotting or mass spectrometry analysis of isolated LBs. Further research into the molecular interactions between p-α-syn and pBRCA1 (Ser1423) as well as the roles of pBRCA1 and BARD1 in different brain regions could shed light on the distinct pathways leading to neurodegeneration in LBD. These findings may provide new insights into the mechanisms of inclusion formation or help in developing therapeutic strategies targeting region-specific vulnerabilities in LBD.
